# Complete Genome Sequence of *Gelria* sp. Strain Kuro-4, a Thermophilic Anaerobe Isolated from a Thermophilic Anaerobic Digestion Reactor Treating Poly(L-Lactic Acid)

**DOI:** 10.1128/MRA.00544-21

**Published:** 2021-08-19

**Authors:** Takeshi Yamada, Masako Hamada, Misaki Kurobe, Takashi Narihiro, Hideto Tsuji, Hiroyuki Daimon

**Affiliations:** a Department of Applied Chemistry and Life Science, Toyohashi University of Technology, Toyohashi, Aichi, Japan; b Bioproduction Research Institute, National Institute of Advanced Industrial Science and Technology (AIST), Sapporo, Hokkaido, Japan; c Student Support Center, Toyohashi University of Technology, Toyohashi, Aichi, Japan; Indiana University, Bloomington

## Abstract

Strain Kuro-4 was isolated as a novel member of the genus *Gelria* from a thermophilic anaerobic digestion reactor treating poly(l-lactic acid). Here, we report a 2,880,462-bp complete circular genome sequence of Kuro-4, with a G+C content of 61.9%. The chromosome harbors 2,831 protein-coding genes and 62 RNA-coding genes.

## ANNOUNCEMENT

To date, Gelria glutamica is the only authentic member of the genus *Gelria* that could utilize amino acids in strictly syntrophic associations with hydrogenotrophic methanogens (1). We successfully obtained a novel *Gelria* isolate (strain Kuro-4) from a thermophilic anaerobic digestion reactor treating poly(l-lactic acid). Here, the complete genome sequence of the previously unknown *Gelria* bacterium was sequenced.

Serial dilution of a roll-tube culture (2) containing l-lactate (1 mM) and yeast extract (0.01%) was used to isolate the strain Kuro-4. A pure culture of strain Kuro-4 was anaerobically cultivated at 55°C in basal medium (3) (pH 7.0) supplemented with pyruvate (10 mM) and yeast extract (0.01%). DNA was extracted using a NucleoBond high-molecular-weight (HMW) DNA kit (TaKaRa Bio Inc., Shiga, Japan) according to the manufacturer’s instructions. For long-read sequencing, genomic DNA was barcoded using a native barcoding expansion kit (EXP-NBD104; Oxford Nanopore Technologies [ONT], Oxford, UK), and the long-read library was adjusted using a ligation sequence kit (SQK-LSK109; ONT). The long-read sequences were determined using the GridION X5 system (ONT) on an R9.4.1 flow cell (ONT), and a total of 164,703 raw reads with an *N*_50_ length of 7,254 bp were base called using Guppy v4.011+f1071ce (ONT). After the adapter sequence was removed using Porechop v0.2.4 (https://github.com/rrwick/Porechop), <1,000-bp reads were discarded using Filtlong v0.2.0 (https://github.com/rrwick/Filtlong). Read errors were corrected using Canu v2.0 (4) with default settings, and a total of 25,681 reads (*N*_50_ length of 11,581 bp) was obtained. A short-read library was prepared using fragmented DNA and an easy universal DNA library prep set (MGI Tech, China). Circularized DNA was prepared using an easy circularizing kit (MGI Tech), and paired-end sequences (2 × 200 bp) were determined using a DNBSEQ-G400RS high-throughput sequencing kit (MGI Tech) and the DNBSEQ-G400 platform. The adapter sequences of 15,180,682-bp paired-end sequences were removed using Cutadapt v2.10 (5), and 3.5 million read pairs were sampled using Seqkit v0.12.0 (6). Among the sequences, bases with a quality score of <20 were removed using Sickle v1.33 (https://github.com/najoshi/sickle) and reads with <127 bases and their pairs were discarded. For long-read and short-read hybrid assembly, Unicycler v0.4.9 (7) was used with default settings. The completeness of the genome sequence was verified using CheckM v1.0.7 (8). The complete genome sequence was annotated using the DDBJ Fast Annotation and Submission Tool (9) and functionally annotated using the Kyoto Encyclopedia of Genes and Genomes database (10).

The final chromosome sequence was 2,880,462 bp long with 1,274-fold average coverage and G+C content of 61.9%. The genomic sequence consisted of 2,831 protein-coding genes and 62 RNA-coding genes, including 9 rRNA and 52 tRNA genes and 1 transfer-mRNA gene. In addition, genes encoding enzymes associated with the *Rhodobacter* nitrogen fixation complex and ATP synthetase were also identified. This complete genome sequence is the first reported whole-genome information for a member of the genus *Gelria*.

### Data availability.

This complete genome sequence has been deposited at DDBJ/GenBank/EMBL under the accession number AP024619. Raw read sequences are available in NCBI under the Sequence Read Archive accession number DRA011334.
